# The relationship between social withdrawal and problematic social media use in Chinese college students: a chain mediation of alexithymia and negative body image

**DOI:** 10.1186/s40359-024-01755-0

**Published:** 2024-05-03

**Authors:** Shuang Li, Xiaomei Chen, Lili Liu, Chongyong Sun

**Affiliations:** 1 Jilin Normal University, Siping, China; 2 LuDong University, Yantai, China; 3 Northwest Normal University, Lanzhou, China; 4 School of Educational Science & Institute of Psychology, Jilin Normal University, Siping, 136000 Jilin Province China

**Keywords:** Social Withdrawal, Problematic social media use, Alexithymia, Negative body image, College students

## Abstract

This study explores the relationship between social withdrawal and problematic social media use among college students, with a focus on the mediating roles of alexithymia and negative body image. Using the University Student Social Withdrawal Questionnaire, Social Media Addiction Scale, Toronto Alexithymia Scale, and Negative Body Image Scale, 2582 college students (33.46% male, average age = 19.46 years, *SD* = 2.23) were surveyed. Social withdrawal, alexithymia, negative body image, and problematic social media use were significantly correlated with each other. Social withdrawal positively predicted problematic social media use, and both alexithymia and negative body image played a chain mediating role between social withdrawal and problematic social media use. The findings indicate that individual social withdrawal is associated with college students’ problematic use of social media. The results suggest that alexithymia and negative body image may mediate this association, highlighting a potential pathway through which social withdrawal influences social media use patterns.

## Introduction

According to the 50th Statistical Report on the Development of the Internet in China released by the China Internet Network Information Center (CNNIC) in Beijing, as of June 2022, China’s internet users numbered 1.051 billion, with 1.047 billion accessing the internet via mobile devices, accounting for 99.6% of the total internet user base. This widespread accessibility has been paralleled by continuous innovations in social media products. Features such as audio content, short videos, and live streaming have gained immense popularity, enhancing the ecosystem of social networking sites. These innovations not only meet users’ personalized demands but also significantly enrich the social media experience.The diversification of social media functions plays a dual role. Firstly, it caters to a wide range of user interests and preferences, encouraging engagement through personalized content and interactive features. This personalization fosters a sense of connection and community among users, enhancing their overall experience. Secondly, the multitude of available functions—including gaming, shopping, and social comparisons—has been shown to increase the time users spend on social media platforms. This increased engagement contributes to higher ‘stickiness’, a term used to describe users’ propensity to return to and spend time on the platform.

However, while these features can offer positive experiences, they also present potential risks. The ease of access to such a wide array of content can lead to excessive use, where the boundary between healthy engagement and problematic social media use becomes blurred. Problematic social media use, characterized by excessive concern over social media, compulsive need to access or use social media, and difficulty in disengaging, can have adverse effects on individuals’ mental health and well-being. Therefore, while the continuous innovation of social media products has undoubtedly improved the digital social landscape, it also necessitates a critical examination of how these advancements may contribute to the emergence and escalation of problematic social media use among users. Drawing from the understanding of problematic internet use, problematic social media use should be examined within a motivational framework, focusing on the underlying motives and needs of individuals, rather than solely on their behaviors [1]. Problematic social media use can be defined as an individual’s behavioral patterns on social media platforms that exceed the bounds of normal usage, leading to significant adverse effects on their social, psychological, or physical health. This usage pattern is often driven by attempts to fulfill specific psychological needs such as seeking a sense of belonging, self-affirmation, or emotional regulation. However, these attempts are achieved in counterproductive ways that can result in detrimental effects on the individual’s daily functioning and well-being. The core characteristics of problematic social media use include excessive use, emotional dependency, social impact, withdrawal symptoms, functional impairment, and persistent use. Many studies have found that problematic social media use is detrimental to students’ current and future healthy development, causing individuals to feel physiological and psychological discomfort [2]. Students with social media addictive behaviors have poor self-control [3] and are more likely to face academic difficulties [4]. Given the continuous growth and harmfulness of problematic social media use, the academic community has been focusing on the influencing factors and mechanisms of problematic social media use for many years [5]. This study aims to examine the relationship between social withdrawal and college students’ problematic social media use, focusing on the potential mediating roles of alexithymia and negative body image within this context. By doing so, it endeavors to provide empirical support for interventions addressing problematic social media behaviors among college students.

Based on Maslow’s Hierarchy of Needs Theory, Attachment Theory, and the Dual-system model of social cognition, we have constructed a theoretical model of behavior and motivation. This model seeks to explore the relationship between problematic social media use, social withdrawal, alexithymia, and negative body image. Our model begins with Maslow’s Hierarchy of Needs Theory, positing that individuals, after satisfying basic physiological needs, seek fulfillment of needs for safety, love and belonging, esteem, culminating in self-actualization [6]. In the context of contemporary social media, the pathways to satisfying these needs have evolved, particularly those for love and belonging and esteem, which are frequently pursued through online interactions. Subsequently, we incorporate Attachment Theory to explain how individuals’ attachment styles, formed from early relational experiences, influence their behavior on social media. Individuals with secure attachment may exhibit healthier social media use habits, whereas those with anxious or avoidant attachment may be more prone to problematic use. Moreover, we employ Dual-system model of social cognition to analyze how impulse control and self-regulation play roles in social media use. The interaction between System 1 (automatic, unconscious thought processes) and System 2 (conscious, logical thought processes) [7] elucidates why individuals might exhibit excessive social media use under certain circumstances.

Building on this theoretical framework, our paper aims to delve into the psychological underpinnings behind problematic social media use and analyze how social withdrawal, alexithymia, and negative body image serve as mediating variables that bridge the gap between individuals’ basic needs and problematic social media use. Social withdrawal may stem from unmet needs for belonging and insecure attachment styles, leading to difficulties in forming relationships in real life. This might push individuals towards social media as an alternative means of fulfilling their need for belonging, which may not adequately provide genuine social satisfaction, thus leading to problematic use. Alexithymia, as a barrier to understanding and expressing emotions, might be related to early attachment experiences and unmet esteem needs [8]. This barrier could leave individuals feeling helpless in the face of real-life emotional challenges, thereby seeking social media as a means of escaping reality and managing emotions, which could foster problematic use over time. The development of a negative body image might be closely linked to social comparison mechanisms on social media. Social Comparison Theory suggests that individuals tend to assess themselves through comparison with others, and social media provides a broad platform for such comparisons [9]. This continuous comparison may lead to dissatisfaction with one’s body image, thereby hindering the fulfillment of esteem needs and potentially exacerbating problematic social media use. By integrating these theories and research findings, our model aims to provide a more comprehensive perspective on the psychological roots of problematic social media use. This research emphasizes the significance of examining social withdrawal, alexithymia, and negative body image as mediating factors in order to elucidate the mechanisms through which individuals engage with social media in attempts to satisfy unmet fundamental needs, and the potential challenges encountered within this context. This understanding can help develop targeted interventions to reduce problematic social media use and promote individuals’ mental health and well-being.

In the digital age, the prevalence of social media platforms has revolutionized the way individuals fulfill their basic psychological needs, including the needs for belonging, esteem, and self-actualization, as proposed by Maslow. While these platforms offer unprecedented opportunities for social interaction, they also present unique challenges, contributing to the emergence of problematic social media use. Individuals cannot separate from the need for social interaction during the process of socialization. Interpersonal communication plays a unique role in the process of individual socialization. Social withdrawal, as one of the internalized problematic behaviors in social interactions, is manifested in the individual’s external behavioral inhibition and restraint. It also includes the lack of internal interaction motivation, a tendency towards loneliness temperament, and experiences of shyness, collectively manifesting as overall negativity, feelings of loneliness, and peer exclusion [10]. Existing research has pointed out that individuals with social withdrawal behaviors are more likely to experience poor interpersonal relationships and difficulties in social adaptation [11]. In schools, social withdrawal behaviors are significantly correlated with individual peer acceptance, peer rejection, and bullying within the class [12, 13]. The physiological symptom enhancement model suggests that social withdrawal behaviors may be related to the physiological symptoms and responses individuals experience during social interactions. These symptoms and responses might physiologically amplify the individual’s tendency to withdraw [14]. Social situations may trigger the activation of an individual’s autonomic nervous system, such as an accelerated heart rate, sweating, and trembling. These physiological responses could cause individuals to feel discomfort and tension, thereby exacerbating their social anxiety and prompting them to avoid social interactions [15]. Social withdrawal, as a significant way to alleviate social pressure, is likely to exacerbate the risk of problematic social media use. Overseas research has provided supportive evidence. Cross-sectional studies have found that social withdrawal is a risk factor inducing addictive behaviors towards social media [16–18]. Longitudinal studies also indicate that social withdrawal is closely related to students’ problematic behaviors [19]. Therefore, based on prior evidence, This study suggests that social withdrawal serves as a key factor associated with problematic social media use, with this relationship being mediated by the presence of alexithymia and negative body image. Social withdrawal, characterized by behavioral inhibition and a lack of motivation for real-life interactions, often stems from unfulfilled needs for belonging and poor peer relationships. Such withdrawal can lead individuals to seek out digital spaces as alternative venues for social interaction, albeit with potentially maladaptive outcomes.

In refining our discussion on alexithymia and its role in problematic social media use, it’s crucial to integrate the diverse theoretical frameworks that have explored the development of alexithymic traits. This comprehensive examination not only deepens our understanding of alexithymia but also highlights its complexity as a condition not yet fully understood within the psychological community. Alexithymia, characterized by an individual’s marked difficulty in identifying and expressing emotions, alongside a diminished imaginative capacity, encompasses challenges in recognizing subjective emotional states and a limited ability to communicate these feelings to others. This condition is seen as a significant barrier to emotional processing [20]. Investigating alexithymia through the lenses of attachment theory, psychoanalysis, and self-determination theory provides insightful perspectives into the genesis and manifestation of alexithymic traits, especially regarding social interactions and personal relationships. Attachment theory suggests that the nature of early attachment relationships significantly shapes one’s capacity for emotional processing and expression. Insecure attachments, formed during childhood, may impede emotional awareness and expression, thereby fostering the development of alexithymic traits. This linkage between attachment styles and alexithymia underscores the pivotal role of early interpersonal experiences in sculpting the emotional dimension of individuals [21]. From a psychoanalytic perspective, Taylor & Bagby (2013) present alexithymia as a construct that can be deciphered through an exploration of unconscious processes and defense mechanisms, suggesting that alexithymia may emerge from a complex interaction between an individual’s psychological defenses and their emotional processing capabilities [22]. Furthermore, the application of self-determination theory to alexithymia underscores the significance of psychological needs and autonomy in cultivating emotional competencies. Environments that foster autonomy and meet basic psychological needs are essential for the healthy development of emotional awareness and regulation. In contrast, environments that thwart these needs may pave the way for the development of alexithymic traits, highlighting the importance of supportive contexts in reducing the risk of alexithymia [23]. Individuals with pronounced alexithymia often face challenges in emotional regulation and may engage in addictive behaviors within social contexts as a means to manage their emotions. The compensatory use of social media emerges as a response to their difficulties in processing and expressing emotions in real-life scenarios. The dual-system model of social cognition suggests that the immediacy of social media creates an environment conducive to habitual use, with individuals turning to social media during moments of emotional discomfort or loneliness. Research indicates that alexithymia is linked to comorbid disorders associated with social withdrawal, such as internet dependency, internalizing disorders, and social issues [24, 25], thus potentially heightening the propensity for social media addiction among socially averse adolescents [26]. Attachment theory [27] elucidates how early relational patterns with caregivers shape future social interactions and self-perception, with emotional traumas and the lack of secure attachment adversely affecting one’s emotional expressiveness and processing abilities. This predisposition may drive individuals towards alternative forms of intimacy and support systems, such as social media, fostering overuse and dependency. Empirical evidence suggests that alexithymia can predict future internalizing behavioral issues in students [28].

Negative body image, a significant concern in interpersonal dynamics, emerges as a pivotal element in the development of social anxiety [29]. It encompasses an individual’s critical perspective on their physical appearance, characterized by dissatisfaction, negative evaluation, and adverse interpretation [30, 31]. Rooted in Social Comparison Theory, individuals engage in comparisons with others to gauge their abilities and self-worth. This comparative process is particularly intensified among those who are socially withdrawn, as they may primarily interact through social media, exacerbating the frequency and intensity of negative social comparisons. The relentless exposure to idealized and altered images on these platforms can magnify their body dissatisfaction [32], with a lack of positive real-life reinforcement further solidifying this negative self-view. For individuals grappling with social withdrawal, social media platforms can serve as a double-edged sword, providing temporary solace or positive feedback, yet potentially fostering increased dependency and elevating the risk of addiction [33]. From the perspective of reinforcement learning, if a behavior produces a positive outcome, it’s more likely to recur in the future [34]. Studies suggest that for those socially withdrawn or with a negative body image, such escapism provides momentary relief and is seen as a positive reinforcement. When a behavior consistently receives positive reinforcement, it could lead to dependency, culminating in problematic social media use behavior [35–37]. Thus, in light of the aforementioned evidence, The research indicates that negative body image acts as a mediating variable in the relationship between social withdrawal and problematic social media use. Although alexithymia and negative body image are acknowledged as predictors of problematic social media use [38], the precise pathways through which they interact and influence the consequence of social withdrawal on such behavior require additional investigation. Furthermore, studies have indicated that individuals with alexithymia face challenges in recognizing and processing emotions related to body image, which can exacerbate negative perceptions of their bodies and drive them towards social media for emotional gratification [39]. The co-occurrence of emotional dysregulation and negative body image may lead to an overreliance on social media for fulfilling unmet social and emotional needs, thus increasing the propensity for problematic use [40]. social withdrawal may serve as a contributing variable, enhancing the association between alexithymia and negative body image with problematic social media use.

Based on the literature review and theoretical framework, this study posits three hypotheses to investigate the interplay between social withdrawal, alexithymia, negative body image, and problematic social media use. Hypothesis 1: Alexithymia acts as a mediating variable in the relationship between social withdrawal and problematic social media use, with social withdrawal influencing alexithymia, which in turn increases the likelihood of problematic social media use. Hypothesis 2: Negative body image mediates the relationship between social withdrawal and problematic social media use. Hypothesis 3: Social withdrawal indirectly shapes problematic social media use through the chained mediation effects of alexithymia and negative body image.

## Method

### Participants and procedure

Using cluster sampling, we selected 2600 university students from 2 public universities in Jinan and Haikou cities, China, as participants. Before distributing the electronic questionnaire, each participant signed an informed consent form. After removing invalid questionnaires, a total of 2582 valid questionnaires were collected, with an effective response rate of 99.31%. The average age of the students was 19.46 years (SD = 2.23). Among them, males account for 33.46%, while females account for 66.54%. First-year university students make up 68.94%, second-year students make up 19.05%, third-year students make up 8.64%, and fourth-year students make up 3.37%. Single children account for 22.93%, while non-single children account for 77.07%. Urban residents account for 33.86%, while rural residents account for 64.14%. For majors, 30.21% are in Education, 22.70% are in Management, 19.29% are in Law, 8.52% are in Medicine, and 19.28% are in other fields. As for preferred social media, WeChat accounts for 72.67%, Douyin for 11.50%, QQ for 6.74%, and others for 9.49%.

Data collection took place during regular class hours, facilitated by school psychologists. Prior to the measurements, students’ informed consent and passive informed consent from the participants’ parents were obtained. The students were informed that they could withdraw at any time if they felt uncomfortable. All collected information would be used exclusively for scientific research and strictly confidential. All materials and procedures were approved by the ethics committee of the first author’s university and the authoritative bodies of the participating schools. The approval was granted by Ethics Committee of Jilin Normal University on July 15, 2023, under approval number 2023-07-X104. The data collection period spanned from July 2023 to August 2023.

### Measures

#### Demographic questionnaire

We used a self-compiled demographic questionnaire to survey: Gender, Grade/Year level, Only child status (Yes/No), Place of origin (urban/rural), Major/Field of study, Which social network do you prefer? How many contacts (or friends) do you have on the social website you use most frequently?

#### University Student Social Withdrawal Questionnaire

The University Student Social Withdrawal Questionnaire, developed by Tian Yuan in 2012, is designed specifically for adolescents. It comprises 16 items across three dimensions: avoidance of unfamiliar environments, social isolation, and avoidance of public speaking. Responses are captured on a 5-point Likert scale, from 1 (completely disagree) to 5 (completely agree), with higher scores indicating greater levels of social withdrawal. The reliability coefficients for the subscales are as follows: for avoidance of unfamiliar environments, it is 0.705; for social isolation, 0.791; and for avoidance of public speaking, 0.872 [41]. In this study, the overall Cronbach’s alpha for the scale was found to be 0.960, indicating excellent internal consistency .

#### Toronto Alexithymia Scale (TAS-20)

The Toronto Alexithymia Scale (TAS-20) is a widely used self-report measure designed to assess alexithymia. It comprises 20 items across three subscales: difficulty describing feelings (DDF), difficulty identifying feelings (DIF), and externally-oriented thinking (EOT). The scale utilizes a 5-point scoring system, with responses ranging from 1 (strongly disagree) to 5 (strongly agree). A cumulative score is calculated, with respondents scoring 61 or above classified as having alexithymia, scores of 51 to 60 indicating possible alexithymia (borderline), and scores of 50 or below suggesting the absence of alexithymia. A higher score indicates a more pronounced presence of alexithymic traits [42]. The reliability coefficients for the subscales are as follows: 0.825 for DDF, 0.834 for DIF, and 0.852 for EOT. In this study, the overall Cronbach’s alpha for the scale was 0.809, indicating good internal consistency.

#### Negative body image scale

The Scale used in this study was developed by Chen Hong and revised by Liu Daqing (2009), consisting of 27 items. The scale encompasses four dimensions: overall satisfaction, height, appearance, and weight, with 3, 9, 7, and 8 items respectively. The scale uses a 5-point scoring system, from 1 (completely disagree) to 5 (completely agree). A higher score indicates greater dissatisfaction with one’s body image [43]. The reliability coefficients for the subscales are: 0.865, 0.845, 0.817, and 0.835. The Cronbach’s alpha coefficient for this scale in the study was 0.916.

#### Social Media Addiction Scale

In our study, the assessment of problematic social media use among college students was conducted using the Social Media Addiction Scale. The Scale, developed by Liu (2019) and colleagues, was used to assess the degree of problematic social media use among college students. The scale consists of 28 items, divided into six factors: preference for online social interaction, mood alterations, negative consequences and continued use, compulsive use and withdrawal, salience, and relapse. There are no reverse-scored items. The scale employs a 5-point scoring system, from 1 (completely disagree) to 5 (completely agree). A higher total score suggests a higher degree of problematic social media use [44]. The reliability coefficients for the factors are: 0.835, 0.836, 0.828, 0.824, 0.835, and 0.736. The Cronbach’s alpha coefficient for this scale in the study was 0.967.

## Results

### Data processing and common method bias test

Given that all data in this study are self-reported by respondents, it’s essential to control and inspect for common method biases. In the distributed questionnaire, some items were reverse-scored, and several unrelated questions were set up to control the influence of common method bias during data collection. SPSS 25.0 was used for data input and to conduct descriptive statistical analysis and correlation analysis. A structural equation model was constructed using Amos 28.0.The SEM was constructed using the Maximum Likelihood (ML) estimation method. This method is widely used for its robustness in handling complex models and its ability to provide accurate parameter estimates and standard errors under a variety of conditions. The Harman single-factor test was employed to examine the common method bias. The results indicated that the explained variance of the first factor is 33.21%, which is less than the 40% critical value, suggesting that there isn’t a significant common method bias in the study’s data [45].

### Gender differences test for various variables

Upon importing the dataset into SPSS, an analysis employing independent samples t-tests was conducted to examine gender disparities across several variables. The analysis yielded no statistically significant difference in negative body image between male and female college students (*p* > 0.05). Conversely, significant gender differences were observed in the domains of social withdrawal, alexithymia, and problematic social media use (*p* < 0.05), with female students demonstrating higher mean scores in comparison to their male counterparts for each of these variables ( Table 1).

**Table 1:** Gender Differences Test (*t*) for Various Variables Among College Students

Variable	Male	Female	t	|Cohen’s d|
	M ± SD	M ± SD		
Social withdrawal	41.89 ± 15.50	46.64 ± 14.02	-7.83^***^	0.32
Alexithymia	54.21 ± 10.36	55.11 ± 9.32	-2.24^*^	0.09
Negative body image	70.19 ± 18.03	71.43 ± 18.85	-1.60	0.07
Problematic social media use	81.48 ± 23.59	85.12 ± 17.80	-4.36^***^	0.17

Note. *p < 0.05;**p < 0.01;***p < 0.001

### Descriptive statistics and correlation analysis for each variable

Descriptive statistics and correlation analyses were conducted for social withdrawal, alexithymia, negative body image, and problematic social media use. The results from the Pearson correlation analysis indicate that: Social withdrawal is positively correlated with alexithymia (*r* = 0.598, *p* < 0.001). Social withdrawal is positively correlated with negative body image (*r* = 0.474, *p* < 0.001).Social withdrawal is positively correlated with problematic social media use (*r* = 0.435, *p* < 0.001).These findings are consistent with previous theoretical analyses. The other variables also exhibit positive correlations with each other, indicating that the data is suitable for subsequent structural equation model analyses ( Table 2).

**Table 2:** Descriptive statistics and correlation analysis for each variable (*r*)

Variable	M	SD	1	2	3	4
1Social withdrawal	45.05	14.70	-			
2 Alexithymia	54.81	9.69	0.598***	-		
3Negative body image	71.02	18.59	0.474***	0.552***	-	
4problematic social media use	83.89	20.00	0.435***	0.399***	0.402***	-

Note. *p < 0.05;**p < 0.01;***p < 0.001

### Testing the chain mediation effect of alexithymia and negative body image

**Fig. 1:**
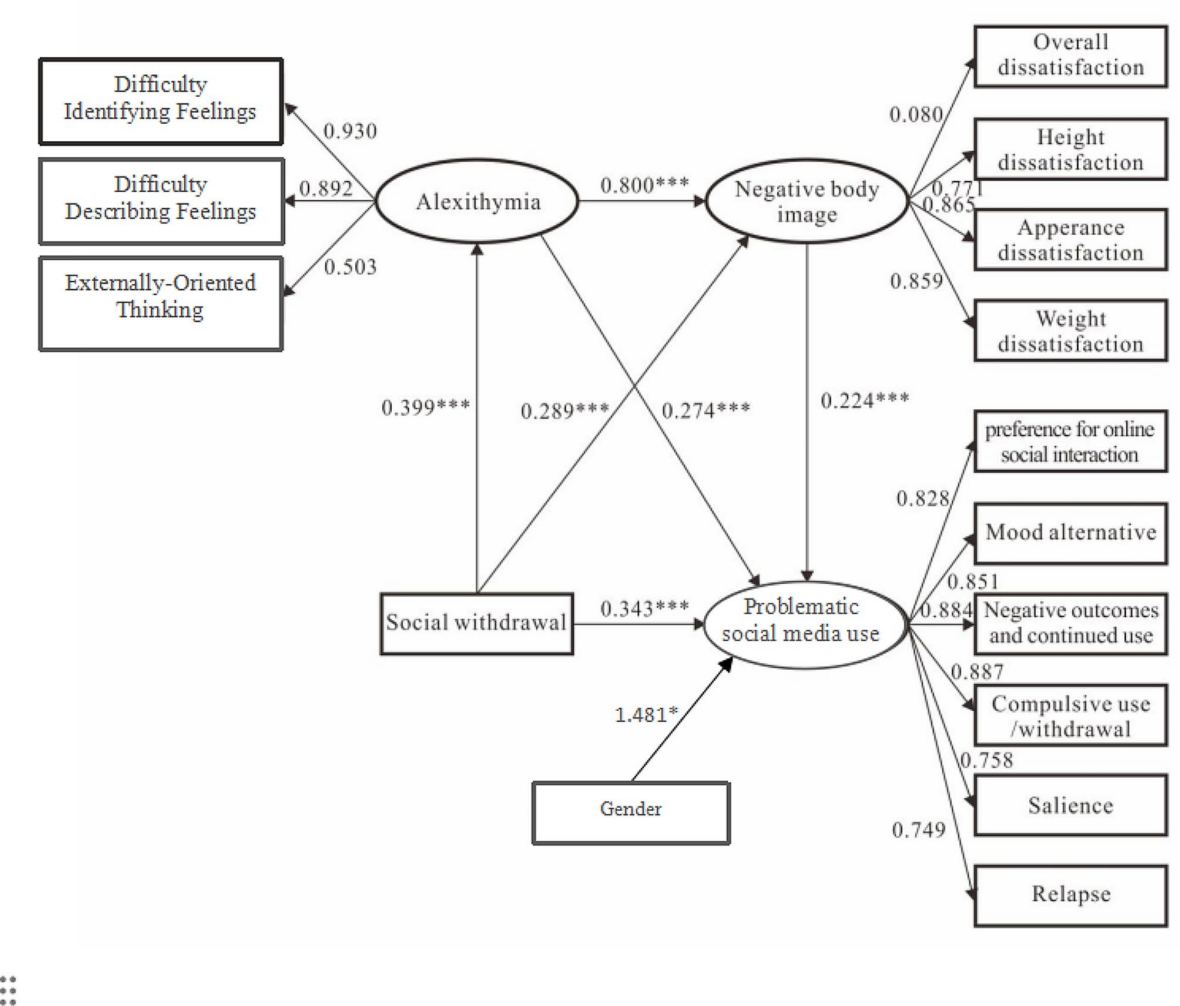
Effect diagram of chain intermediary model. Note: *p < 0.05;**p < 0.01;***p < 0.001

In accordance with the model hypothesis, which posits problematic social media use as the outcome variable, social withdrawal as the independent variable, and alexithymia and negative body image as successive mediating variables, we constructed a chained mediation model for structural equation analysis. To refine our approach and address the influence of gender differences identified in our preliminary analyses, we have incorporated gender as a covariate in the model. From a random sample of 1,300 individuals, the overall model fit indices were: *χ2* = 83.786, *df* = 28, *P* < 0.001, CFI = 0.991, TLI = 0.986, NFI = 0.987, RMSEA = 0.036, indicating a good fit of the model. The results of the structural equation model analysis showed that social withdrawal could positively predict alexithymia (*β* = 0.399, *P* < 0.001), alexithymia could positively predict negative body image (*β* = 0.800, *P* < 0.001), and negative body image could positively predict problematic social media use (*β* = 0.224, *P* < 0.001). This implies that higher scores of social withdrawal lead to increased levels of alexithymia, which in turn exacerbate negative body image, ultimately intensifying behaviors of problematic social media use. All other path coefficients in the model (Fig. 1) reached a significant level, where alexithymia and negative body image played a mediating role between social withdrawal and problematic social media use among college students.

This mediation role consisted of three paths: Path 1: regarding the mediation by alexithymia, the findings indicate that alexithymia partially mediates the relationship between social withdrawal and problematic social media use, with ab = 0.109, 95% CI [0.066, 0.153], validating Hypothesis H1. Path 2: concerning the mediation by negative body image, the results demonstrate that negative body image also plays a partial mediating role in this relationship, with ab = 0.071, 95% CI [0.054, 0.091], supporting Hypothesis H2. Path 3: chained mediation of alexithymia→negative body image, results indicated that alexithymia→negative body image played a chained mediation role in the process of social withdrawal influencing problematic social media use with ab = 0.065, 95% CI [0.048, 0.085], validating Hypothesis H3. The above findings suggest that alexithymia and negative body image indirectly influence the process in which social withdrawal intensifies the risk of problematic social media use among college students. Moreover, alexithymia and negative body image further influenced the risk posed by social withdrawal intensifying problematic social media use through a chained-mediation mechanism. Therefore, Hypotheses 1, 2, and 3 were all supported. Refer to Fig. 1. Details of the mediation effect analysis can be found in Table 3.

**Table 3:** Path and effect values of the intermediary model

Path	Effect	SE	Boot LL CI	Boot UL CI	Relative mediation effect
Social withdrawal-Alexithymia-Social media addiction	0.109	0.022	0.068	0.153	18.57%
Social withdrawal - Negative body image-Social media addiction	0.072	0.009	0.055	0.092	12.27%
Social withdrawal-Alexithymia-Negative body image-Social media addiction	0.065	0.009	0.048	0.084	11.25%
Total indirect effect	0.245	0.023	0.203	0.292	41.74%
Direct effect	0.342	0.030	0.283	0.401	58.26%

## Discussion

### Exploring the gender differences among college students in social withdrawal, alexithymia, and problematic social media use

This study was designed to examine the relationship between social withdrawal and social media use patterns among college students, with an analysis revealing notable gender differences. Female students exhibited higher scores in social withdrawal, alexithymia, and tendencies towards social media use compared to their male counterparts. Eagly posited that gender differences in social behaviors stem from differing gender role expectations [46]. In different cultural contexts, girls are often encouraged to demonstrate qualities of introversion, gentleness, and respect, while boys are nudged towards exhibiting traits of extroversion and confidence. This gender role stereotype prevails across many cultures, including those of China, India, the Middle East, and Western societies. Despite efforts in some regions to promote gender equality and empower women, this stereotype persists globally, exerting profound effects on individuals and societal roles. This socialization might lead to females displaying more social withdrawal behaviors in certain situations [47]. Moreover, in the broader societal context, females, more passive in interpersonal interactions, are less likely to initiate conversations, making them more prone to feelings of shyness and timidity in public social settings [48]. All these factors curtail the social activities of females, leading to gender differences in social withdrawal among college students, consistent with prior research [49].

The study also found that female students had significantly higher scores for alexithymia than males. Several factors could contribute to this phenomenon. societal and cultural norms around gender and emotionality might influence how males and females perceive and report their emotional experiences. Females are often socialized to be more attuned to their emotional states and expressive about their feelings, potentially leading to a higher self-reporting of alexithymia [50]. In contrast, males might underreport such difficulties due to societal expectations that valorize emotional stoicism. Furthermore, the stressors associated with academic and social environments in college could exacerbate emotional regulation difficulties, particularly for females who may face unique pressures related to performance, appearance, and interpersonal relationships. These stressors might lead to heightened vulnerability to emotional expression disorders among female students [51, 52]. Gibbons & Buunk (1999) highlighted that due to society’s heightened focus on females, they might be more attuned to comparing themselves with peers, leading to heightened self-criticism and barriers in emotional expression [53]. As females grow, interactions with peers might expose them to more information on emotional relationships, making them more susceptible to related challenges or barriers [54]. This aligns with the findings of Xu Junjie [55].

Analyzing gender differences in problematic social media use among college students revealed a higher inclination among females. In the context of the ongoing digital evolution, phenomena such as online shopping and virtual communication have gained widespread popularity. Our findings suggest that these activities appeal to a broad audience, including females, though it is important to recognize that engagement with digital platforms varies widely among individuals, transcending simplistic gender categorizations. They might use social media more for social comparisons, especially concerning body image [56]. Shyer females in real life might prefer social media platforms to express themselves and forge social connections. Additionally, they might be more drawn to certain content types and cultures prevalent on social media platforms [57], potentially exacerbating their tendencies for addiction [58].

### Independent mediating roles of alexithymia and negative body image

The study indicates that alexithymia mediate between social withdrawal and problematic social media use. Firstly, there is a direct relationship observed between social withdrawal and the emergence of alexithymia among college students. Such behavior might impose significant stress, especially in situations where persistent concerns about interpersonal interactions exist. This, combined with diminished control and self-regulation, might distract from understanding others’ feelings or behaviors correctly [59]. Following the Dual-System Model of Cognition [60], individuals under such stress might rely on automated reactions over deep contextual analysis. This might lead to misinterpretations, amplifying their emotional expression disorders. Secondly, these disorders can directly predict problematic social media use. The anxiety-avoidance hypothesis [61] suggests that for those experiencing emotional expression disorders, social media might serve as an escape from real-life challenges, gradually leading to addiction.

Results also showed that negative body image mediates between social withdrawal and problematic social media use. Social withdrawal can predict an individual’s negative body image directly. Given the societal emphasis on social interaction, socially withdrawn individuals might often face challenges and barriers [62], leading to feelings of alienation and consequent dissatisfaction with one’s appearance. This aligns with the Spillover Hypothesis, suggesting negative experiences in one domain can “spillover” and influence another [63]. Further, a higher negative body image makes students more susceptible to problematic social media use, consistent with the Behavioral Reinforcement Theory. Those with a negative self-assessment might seek validation online, craving praise or positive feedback to offset real-world insecurities. Social media platforms often cater to these needs through immediate positive feedback like likes or comments, reinforcing continued usage. Thus, negative body image acts as a “bridge” between social withdrawal and problematic social media use.

### Chain mediation role of alexithymia and negative body image

After controlling for the covariate of gender, Our investigation uncovered a significant pathway linking social withdrawal to problematic social media use, mediated by alexithymia and negative body image. The results suggest that alexithymia functions as an independent mediator and also indirectly contributes to problematic social media use through its association with negative body image. Specifically, alexithymia emerges as a distinct risk factor for developing a negative body image. This relationship is supported by the Social Information Processing Model, which posits that individuals’ processing of body-related information is shaped by past experiences and beliefs. Consequently, these emotional processing disorders may heighten dissatisfaction with one’s appearance, prompting increased social media use as a means of seeking external validation [64]. Over time, such dependency may escalate into addiction.In summary, our study highlights the intricate mechanisms by which alexithymia and negative body image mediate the effect of social withdrawal on problematic social media use. Understanding these dynamics offers critical insights for developing targeted interventions aimed at mitigating the risk factors associated with problematic social media use among college students.

### Clinical implications

Considering that college students are at a pivotal stage of personality development and identity formation, our study’s exploration of the link between social withdrawal and problematic social media use offers valuable insights for reducing factors that contribute to problematic usage and, consequently, enhancing students’ mental health. Specifically, we propose offering counseling on social withdrawal as a preventive measure against problematic social media use. This approach can serve as a guide for both families and educational institutions in their practices. Furthermore, by improving education on issues related to dysphoria and negative body image among college students, we can decrease problematic social media use and elevate the awareness of both schools and parents regarding the importance of mental health education for college students.

Clinicians should consider incorporating assessments for social withdrawal, alexithymia, and negative body image when evaluating individuals for problematic social media use. Recognizing these underlying factors is crucial for a holistic treatment approach. Based on our findings, interventions should be tailored to address the specific needs and issues identified. For individuals with significant social withdrawal, strategies to enhance social skills and real-world interactions could be beneficial. For those with alexithymia, emotional awareness and expression training might be incorporated. Addressing negative body image may involve cognitive-behavioral techniques to challenge and reframe negative thoughts about body image.

### Limitations and prospects

This study unveils the intricate mechanisms through which social withdrawal interacts with problematic social media use among college students, elucidating the interconnections between social withdrawal, alexithymia, negative body image, and problematic social media usage. It also offers theoretical insights and practical suggestions for mental health education. Nonetheless, the research is subject to certain limitations arising from the choice of subjects and the research methodology employed. Firstly, the study focuses on college students as its primary subjects. Considering the pathological characteristics associated with narrative disorders and social withdrawal, obtaining more precise data through actual clinical populations is essential for identifying more valid indicators. Future studies should aim to re-validate the findings with clinical adolescent groups across various regions and age groups to enhance the replicability of the results. Secondly, the cross-sectional nature of this study restricts its capacity to capture the dynamic evolution of how social withdrawal interacts with problematic social media use. Longitudinal research methods are recommended for future studies to explore the changing relationship between social withdrawal and problematic social media use over time. Thirdly, the reliance on questionnaire data, despite being supported by relevant theories and previous research findings, poses challenges in establishing causal relationships between variables. This study primarily tests correlations, making it difficult to ascertain causality. Future research could integrate additional methodologies to uncover the causal links between these variables more effectively. Fourthly, while this study examines the potential mediating roles of alexithymia and negative body image, acknowledging that other possible mediating variables may also play a part in the connection between social withdrawal and problematic social media use. Subsequent research could explore these additional mediating variables to provide a more comprehensive understanding of the factors contributing to problematic social media use among college students.

## Data Availability

All data and materials related to this study are publicly available or can be obtained by contacting the corresponding author.
